# Acceptable Inclusion Levels for Selected Brown and Red Irish Seaweed Species in Pork Sausages

**DOI:** 10.3390/foods11101522

**Published:** 2022-05-23

**Authors:** Halimah O. Mohammed, Michael N. O’Grady, Maurice G. O’Sullivan, Ruth M. Hamill, Kieran N. Kilcawley, Joseph P. Kerry

**Affiliations:** 1 Food Packaging Group, School of Food and Nutritional Sciences, College of Science, Engineering and Food Science, University College Cork, T12 K8AF Cork, Ireland; halimahmoh8@gmail.com (H.O.M.); michael.ogrady@ucc.ie (M.N.O.); 2 Sensory Group, School of Food and Nutritional Sciences, College of Science, Engineering and Food Science, University College Cork, T12 K8AF Cork, Ireland; 3 Food Quality and Sensory Science Department, Teagasc Food Research Centre, Ashtown, D15 DY05 Dublin, Ireland; ruth.hamill@teagasc.ie; 4 Food Quality and Sensory Science Department, Teagasc Food Research Centre, Moorepark, Fermoy, P61 C996 Cork, Ireland; kieran.kilcawley@teagasc.ie

**Keywords:** seaweeds, sausages, functional ingredient, nutritional composition, processed meats

## Abstract

Commercially available Irish edible brown (*Himanthalia elongata*—sea spaghetti (SS), *Alaria esculenta*—Irish wakame (IW)) and red (*Palmaria palmata*—dulse (PP), *Porphyra umbilicalis*—nori) seaweeds were incorporated into pork sausages at 1%, 2.5%, and 5%. Proximate composition, salt, water-holding (WHC), cook loss, instrumental colour analysis, texture profile analysis (TPA), and sensory analysis were examined. Protein (13.14–15.60%), moisture (52.81–55.71%), and fat (18.79–20.02%) contents of fresh pork sausages were not influenced (*p* > 0.05) by seaweed type or addition level. The ash content of pork sausages containing PP, SS, and IW at 2.5% and 5%, and nori at 5%, were higher (*p* < 0.05) than the control sample. In comparison to the control, sausages containing nori, SS, and IW at 5% displayed higher (*p* < 0.05) WHC. Cook loss was unaffected (*p* > 0.05) by the addition of seaweeds into sausage formulations, compared to the control and within each seaweed. The addition of seaweeds into sausages had an impact on the surface colour (L* a* b*) and texture profile analysis (TPA) at different inclusion levels. Overall, hedonic sensory acceptability decreased (*p* < 0.05) in cooked sausages containing PP at 2.5% and 5%, and SS and IW at 5%.

## 1. Introduction

Interest in food products that can promote health and well-being has increased within the food industry and amongst consumers. Such foods are generically termed ‘functional foods’ where health benefits exist beyond basic nutrition [1]. Since ancient times, seaweeds have been consumed as food and utilised as traditional drugs in Asian countries. Recently, seaweeds have gained significance in western countries as a functional food or as ingredients for use in the development of functional food products [2,3].

Seaweeds contain bioactive compounds and nutritional properties such as high levels of quality protein, minerals, vitamins, dietary fibre, polyphenols, carotenoids, and tocopherols [4]. They are also low in fat, and selected species contain prominent levels of n-3 polyunsaturated fatty acids (PUFAs) [5]. Bioactivities and health benefits associated with compounds present in seaweed include antioxidant, anti-inflammatory, anti-coagulation, anti-proliferation, and antibiotic properties [6]. In addition to enhancing healthiness, seaweeds can also impact the physicochemical and sensory properties of meat products. Seaweed polysaccharides (dietary fibre) alginate from brown seaweeds, and agar and carrageenan from red seaweeds have been extensively utilised by the food industry as texture modifiers due to their high viscosity and gelling properties [7]. These textural-influencing properties of seaweeds may enhance the texture and acceptability of processed meat products by consumers. For instance, alginate and carrageenan have been used as fat replacers in processed meats to produce healthier products without compromising on the benefits of fat, such as texture and flavour [8,9,10,11,12,13]. Additionally, agar has also been used as a gelling agent by poultry and meat canners, and as meat substitutes [14]. Minerals such as sodium and potassium are present in brown and red seaweeds in high amounts and these minerals can present a saltiness flavour and taste in meat products. Prominent levels of aspartic and glutamic amino acids in seaweeds are responsible for their distinctive umami flavour and taste, which may be desirable to consumers and increase their interest to consume seaweed-containing meat products [15].

Seaweeds have been added to processed meat products as functional ingredients without reducing other product components such as fat, salt, and phosphate levels, for example, sea spaghetti (*Himanthalia elongata*) in beef patties [16], sea tangle (*Laminaria japonica*) in breakfast sausages [17], sea mustard (*Undaria pinnatifida*), green laver (*Ulva spp*.), and *Sargassum fusiforme* in pork patties [18]. This study is similar to previous studies in that it also investigates the functionality of selected seaweeds, but it differs significantly in that it does so while using commercially available Irish seaweeds at three different inclusion levels. Additionally, the nutritional composition, antioxidant activity, technological and thermal properties of the selected seaweeds employed in this study have been analysed and quantified. Results from this study concluded that these brown and red seaweed species have nutritional sources of protein, amino acids, dietary fibre, minerals, and health-promoting bioactive antioxidant compounds, and demonstrated thermal stability at high temperature application [19].

To the best of our knowledge, seaweed species employed in this study which were harvested from the coast of Ireland have not been extensively explored previously as potential functional ingredients in processed meat products at different inclusion levels. Hence, the objective of this study was to investigate the effect of commercially available brown (*Himanthalia elongata*—sea spaghetti, *Alaria esculenta*—Irish wakame) and red (*Palmaria palmata*—dulse, *Porphyra umbilicalis*—nori) Irish seaweed species on the physicochemical and sensory properties of pork sausages. Acceptable inclusion levels of these seaweeds from a sensory perspective were determined.

## 2. Materials and Methods

### 2.1. Chemicals and Reagents

Sodium chloride (NaCl), hydrogen peroxide (H_2_O_2_), boric acid (H_3_BO_3_), hydrochloric acid (HCl), and Sulphuric acid (H_2_SO_4_) were supplied by Sigma–Aldrich Ireland Ltd., Vale Road, Arklow, Wicklow, Ireland.

### 2.2. Raw Materials

Seaweeds were bought from Wild Irish Seaweed, Ennis, Co. Clare, Ireland. According to supplier specification, all seaweeds were air-dried, then dehumidified and milled. They were hand-harvested off the coast of Co. Clare, Ireland, 100% naturally grown and organically certified. Brown *(Himanthalia elongata*—sea spaghetti (SS), *Alaria esculenta*—Irish wakame (IW)) and red seaweed species (*Palmaria palmata*—dulse (PP), *Porphyra umbilicalis*—nori) were used in this research.

Pork oyster muscle and pork backfat were supplied by Ballyburden Meat Processors (Cork, Ireland). Sausage seasoning (Salt, sugar, dextrose, stabiliser (E450, E452), flavour enhancer (E621), preservative (E223, sodium metabisulphite), antioxidant (E301), rusk (wheat flour (calcium carbonate, iron, niacin, thiamin), salt), natural flavourings, rapeseed oil, hydrolysed vegetable protein (soya, maise)) with a total of 58.78% salt was obtained from Redbrook Ingredient Services (Mulhuddart, Dublin 15, Ireland). Rusk (Wheat flour (wheat flour, calcium carbonate, Iron, Niacin, Thiamin), salt (1.2%), raising agent E503) and collagen casing were purchased from Kiernans Food Ingredients Ltd. (Dundalk Road, Carrickmacross, Co., Monaghan, Ireland).

### 2.3. Sausage Production

Fresh pork sausages (5 kg batches) were formulated (Table 1). Sausages were manufactured by mincing the pork meat and backfat through a 4 mm hole plate (TALSABELL SA., Valencia, Spain). Pork oyster, half of the water and the seasoning were mixed for 60 s at high speed using a bowl chopper (Seydlemann Bowl chopper, Burgstallstraße, Aalen, Germany). The backfat and various seaweeds were added and mixed at high speed for 30 s. The other half of the water was added, and the batter was further mixed for 30 s at high speed. Rusk was added and mixed at low speed for 30 s. Sausage batter was stuffed using piston-type sausage filler (Mainca UK Ltd., Horton, Berkshire, UK) into 21 mm diameter collagen casings (Viscofan, Colfan HL, Navarra, Spain) and hand-linked.

### 2.4. Proximate Composition

Protein (Kjeldahl) and ash (muffle furnace at 550 °C) contents were analysed by AOAC Methods 954.01 and 942.05, respectively [20]. Results were expressed as a g/100 g (%) of sample. Moisture and fat contents of sausages were determined using a SMART Trac system (CEM GmbH, Kamp-Lintfort, Germany).

### 2.5. Salt Analysis

The salt content was determined by titration using silver nitrate [21]. Silver nitrate (0.1 N AgNO_3_) solution was standardised against 0.1% sodium chloride (NaCl) solution. Samples were placed in a muffle furnace as described in Section 2.4. Ash was washed into a conical flask with 20 mL distilled water. Then, 2 mL of indicator (potassium chromate and potassium dichromate) was added, and standardised silver nitrate was used to titrate the solution from a clear yellow to an opaque light orange. Blank titration was performed using 20 mL distilled water.

% salt = (Titre for sample (mL) − Titre for blank (mL))/(Mass of sample (g)) × Molarity of AgNO_3_ × 5.844

### 2.6. Water-Holding Capacity (WHC)

The water-holding capacity was measured using the method described by Lianji and Chen [22]. Fresh sausage samples (10 g) were weighed into glass tubes and heated in a water bath for 10 min at 90 °C. After heating, the samples were removed, wrapped in cheesecloth, and placed in 30 mL centrifuge tubes lined with cotton wool at the bottom of each tube. Samples were centrifuged at 13,300× *g* for 10 min at 4 °C. After centrifugation, the cheesecloth was removed, and samples were reweighed. Water-holding capacity was calculated using the following calculation:

% Water-holding capacity = 1 − ((B − A))/((B × M)) × 100

B is the sample’s weight before heating, A is the weight of the sample after heating and centrifuging, and M is the % moisture of the sample.

### 2.7. Cook Loss

Sausage weight was recorded before and after cooking. Sausages were cooked in a Zanussi oven at 180 °C for 22 min, and an internal temperature of 72 °C was reached. Cook loss was calculated using the following equation:

% Cook loss = Raw weight − cooked weight/Raw weight × 100

### 2.8. Instrumental Colour Analysis

The colour of fresh sausage samples was measured on the outer surface according to CIE L*a*b colour system. The samples were allowed to stay at room temperature for about 1 h before measuring. The samples were flattened, and the colour was measured using a Minolta chromameter (CR400, Minolta Camera Co. Ltd., Osaka, Japan) with an 11 mm-diameter aperture and D65 illuminant. The chromameter was calibrated using a white tile (Y = 93.6, X = 0.3130, y = 0.3193).

### 2.9. Texture Profile Analysis (TPA)

Texture profile analysis was carried out at room temperature using a texture analyser TA-XF10S1.5i (Stable Micro System, Godalming, Surrey, UK) based on a method described by Bourne [23]. Cooked sausage samples were cut into cylindrical slices (10 mm thickness), and were subjected to a two-cycle compression using a 25 kg loading cell. The samples were compressed to 40% of their original heights with a cylindrical probe (SMP/35 compression plate) at a crosshead speed of 1.5 mm/s. The determined factors include hardness (N), the maximum force required for the first compression; adhesiveness (N), the negative force area for the first bite representing the work necessary to pull the compressing plunger away from the sample; springiness (mm), distance sample recovers after initial compression; cohesiveness (Dimensionless), the ratio of positive force area during the second compression; chewiness (N*mm), the product of gumminess and springiness.

### 2.10. Sensory Acceptance Testing (SAT)

Sausages were cooked as described in 2.7 above. Sausages were left to cool to room temperature and cut into cylindrical slices (~2 cm) and labelled with a three-digit random number. The Hedonic (liking) test was carried out using untrained panellists (*n* = 25) who were familiar with pork sausages [24]. Sensory analysis was conducted in sensory booths that conform to international standards [25] at room temperature. Assessors were presented with 13 treatments duplicated in a randomised order over three sessions (first session—10 samples, second and third sessions—8 samples per session) to reduce panellist fatigue. Water for palate cleansing between samples was also provided. Assessors were asked to indicate their scores for the following hedonic terms: appearance, aroma, flavour, texture, and overall acceptability on a 10-cm continuous line scale (1 = extremely dislike and 10 = extremely like). Assessors were also asked to select their most preferred sample. The mean values for each treatment per panellist were analysed. All subjects gave their informed consent for inclusion before they participated in the sensory study. The study was conducted in accordance with the Declaration of Helsinki, and the protocol was approved by university college cork social Research and Ethics Committee (Log 2021-188).

### 2.11. Statistical Analysis

Physiochemical analyses were carried out on three independent trials, on duplicate samples per trial, and three readings were taken per sample. Data were presented in triplicate (average per trial). For SAT data, the mean of each panellist was presented (*n* = 25). Statistical analysis was carried out using the IBM SPSS statistics 25 for windows (SPSS, Chicago, IL, USA) software package. One-way ANOVA was used to compare means of the data obtained from physiochemical and SAT analyses. Tukey’s post-hoc test was used to adjust for multiple comparisons between treatment means at a 5% significance level.

## 3. Results and Discussion

### 3.1. Proximate Composition, Salt, WHC, and Cook Loss

Protein (13.14–15.60%), moisture (52.81–55.71%), and fat (18.79–20.02%) contents of fresh pork sausages were not influenced by seaweed type or addition level (Table 2). Seaweeds employed in this study contained protein (5.57–32.03%), moisture (8.99–16.31%), and fat (1.02–2.03%) contents, but at the inclusion levels employed in this study (1%, 2.5% and 5%), did not significantly impact upon sausage composition [19]. Kim et al. [17] reported similar results in breakfast sausages with added sea tangle at increasing inclusion levels and decreasing pork meat content.

However, the ash content of pork sausages containing PP, SS, and IW at 2.5% and 5%, and nori at 5%, were higher (*p* < 0.05) than the control sample. Pork sausages containing brown seaweeds SS and IW had significantly (*p* < 0.05) higher ash content and these levels increased further with increasing seaweed addition. This observation corresponds with published results observed for sea tangle, a brown seaweed which also had been incorporated into breakfast sausages [17]. From our previous study on these seaweeds, brown seaweeds have higher ash content than the red seaweeds, hence the increasing ash content with increasing brown seaweed addition [19]. Additionally, pork sausages containing red seaweeds PP and nori at 5% had higher (*p* < 0.05) ash content than those containing 1% and 2.5% (Table 2). Mohammed et al. [19] reported the ash content of these red seaweeds to range from 17.2–25.6% and thus showed no significant difference in either ash content between sausages formulated to contain 1% and 2.5% red seaweeds (Table 2). Sausage samples SS5%, IW5%, and PP5% had higher (*p* < 0.05) salt contents than the control products; however, sausage samples containing nori at 5% did not significantly differ to the control. The salt content of these seaweed-containing sausages was contributed directly by the addition of seaweeds, as had previously been quantified by Mohammed et al. [19].

In comparison to the control, sausages containing SS and IW at 2.5 and 5%, nori at 5% displayed higher (*p* < 0.05) WHC, and PP at 1% had a lower (*p* < 0.05) WHC. No significant difference was observed within SS and IW seaweed sausage treatment (Table 2). However, sausages containing PP and nori at 5% had higher (*p* < 0.05) WHC than sausages containing these seaweeds at 1%. Sausages formulated with PP held less water than other seaweed sausage treatments at the different inclusion levels. Mohammed et al. [19] also reported the WHC of SS, IW, and nori to be greater than that of PP. Cook loss was unaffected (*p* > 0.05) by the addition of seaweeds into sausage formulations when compared to the control and within each seaweed (Table 2). Sausage samples significantly lost the same amount of moisture and fat during cooking as samples contained similar water and fat contents (Table 1) and underwent an identical cooking process.

### 3.2. Colour

The surface lightness (L*) values of fresh sausages containing PP and IW at 2.5% and 5%, nori at 1%, 2.5%, and 5%, and SS at 5%, were lower (*p* < 0.05) than those observed for control sausage samples. Kim et al. [17] reported comparable results, in which seaweed sausages had decreased lightness (L*) in comparison to the control due to the dark colour of seaweeds. Surface redness (a*) values decreased (*p* < 0.05) in all seaweed-containing sausage samples, relative to the control, except for sausages containing nori at 1%. The addition of IW at levels of 2.5% and 5% increased the greenness (−a* values) of sausages. Kim et al. [17] also reported negative a* values for sausages containing higher brown seaweed content (3% and 4%). This may be due to the higher contents of carotenoids such as fucoxanthin and chlorophyll c in some brown seaweeds that are responsible for their green colour [26,27]. Similar to the lightness (L*), the yellowness (b*) values decreased (*p* < 0.05) in sausages containing PP and IW at levels of 2.5% and 5%, and nori at 1%, 2.5%, and 5%.

Additionally, within each seaweed species treatment, sausages containing SS at 5% had significantly lower a* values compared to samples containing SS at 1%. The L* value of IW pork sausages decreased (*p* < 0.05) with increasing seaweed addition, 1% IW sausages had higher (*p* < 0.05) a* values than the other seaweed concentrations, and higher (*p* < 0.05) b* value than the 5% IW-containing sausages. Pork sausages containing PP at 1% had higher (*p* < 0.05) L* and b* values than the 5% treatment. Sausages containing 5% nori had lower (*p* < 0.05) L* values than the other inclusion levels and 1% nori sausages had higher (*p* < 0.05) a* and b* values than the 5% nori-containing sausages. Although not significant, with increasing seaweed addition, L*, a*, and b* values decreased (Table 3). Colour changes in pork sausages were attributed to various pigments present in brown and red seaweeds such as phycobiliproteins, carotenoids, and chlorophyll [28].

### 3.3. Texture Profile Analysis (TPA) of Cooked Pork Sausages

The texture profile of cooked pork sausages containing brown and red seaweeds is shown in Table 4. In comparison to the control, texture profile analysis (TPA) results indicated that hardness increased (*p* < 0.05) in sausages containing nori and IW at 5%. Springiness values for sausages containing PP at 2.5% and 5% decreased (*p* < 0.05) compared to the control. No significant differences were observed in relation to seaweed-sausages’ adhesiveness and cohesiveness values in comparison to the control. The addition of nori seaweed at 5% increased (*p* < 0.05) the chewiness of sausage samples when compared to the control.

Within each seaweed species treatment, sausages incorporated with 1% PP were harder (*p* < 0.05) than that of 5% PP, and sausages containing 5% nori and IW were harder (*p* < 0.05) than sausages containing 1% and 2.5% of these seaweeds. Although not significant, increasing addition of PP resulted in the reduction of hardness, springiness, and chewiness values in sausage samples. However, the incorporation of SS, IW, and nori increased the values of hardness and chewiness. Kim et al. [17] reported higher hardness values in sausages with increasing sea tangle included and demonstrated no effect on product cohesiveness.

Brown and red seaweed species contain dietary fibres such as galactans, agar, carrageenan, alginate, fucans, and laminarans, all of which have gelling and thickening properties and can influence the textural properties of pork sausages [29,30]. The type and quantity of these dietary fibres present in these seaweeds may be responsible for the reduction in TPA (hardness, springiness, and chewiness) values with increasing PP addition and an increment in TPA (hardness, springiness, and chewiness) with increasing SS, IW, and nori addition. The addition of seaweeds into meat products, such as patties and sausages, has also been reported to increase hardness values [17,18].

### 3.4. Sensory Analysis of Cooked Pork Sausages

**Figure 1:**
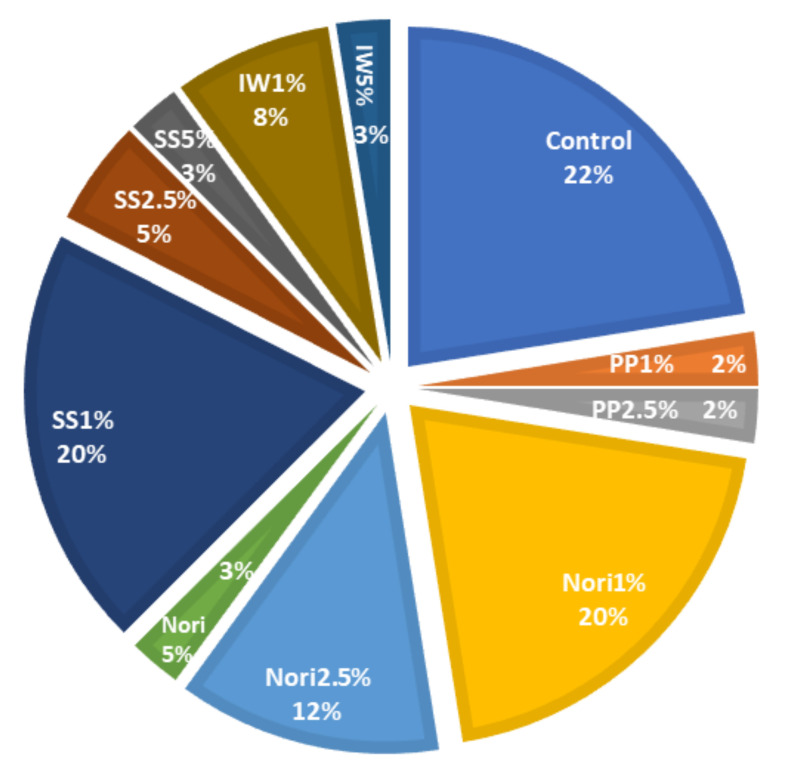
Pie chart representing consumer (*n* = 25) preference.

The sensory results for control and seaweed-containing sausages are shown in Table 5. In comparison to the control, sensory analysis indicated that inclusion of nori and IW at 5% negatively influenced the liking of cooked pork sausage appearance. Sausage aroma scores also decreased (*p* < 0.05) in cooked sausages containing PP, SS, and IW at a 5% inclusion level. Liking of texture was not significantly affected by the addition of seaweed species. Addition of PP at 2.5% and 5%, SS and IW at 5% decreased (*p* < 0.05) flavour scores for cooked pork sausages. Overall sensory acceptability decreased (*p* < 0.05) in cooked sausages containing PP at 2.5% and 5%, and SS and IW at 5%. From instrumental colour analysis, brown and red seaweeds influenced the colour of pork sausages. From previous studies, the liking and perception of seaweed-containing meat product appearance (colour) by consumers were reduced in comparison to the control [17,31]. However, consumers liking of appearance of seaweed-containing sausages in this study were comparable to that of the control. Additionally, food product appearance (colour) is not as relevant to consumers who purchase products based on a perceived healthiness associated with it [32]. It can also be speculated that consumers in this study were more familiar with the consumption of seaweed and seaweed products, and hence, their liking towards these products was positive. Consumers’ (*n* = 25) preferences for sausage treatments are shown in Figure 1 and they followed the order: control > nori 1% ≥ SS1% > nori 2.5% > IW 1% > SS 2.5% > SS 5% ≥ nori 5% ≥ IW 5% > PP 1% ≥ PP 2.5%.

## 4. Conclusions

Addition of seaweeds did not exert significant effects on the proximate composition of fresh pork sausages. However, addition of seaweeds (nori, SS, and IW) at a 5% inclusion level and PP at 2.5% and 5% negatively influenced the colour, instrumental textural properties, and sensory attributes of pork sausages. Colour changes in pork sausages were dependent on the type and quantity of pigments present in seaweeds and the inclusion level of seaweeds used.

Regardless of the impact of these seaweeds on the colour of the sausage samples, panellists’ liking of appearance was comparable to that of the control. From the sensory results of seaweed sausages, consumers preferred sausages with lower seaweed content and liking of seaweed sausages were in the order nori > SS > IW > PP. This study demonstrated that, in pork sausages, a maximum acceptable inclusion level for PP is 1% and 2.5% for the other seaweed species examined. Future research will focus on enhancing the healthiness, such as salt and fat reduction of processed pork products, utilising seaweed species at the acceptable inclusion levels determined in the present study.

## Figures and Tables

**Table 1:** Formulation (%) of pork sausage treatments.

Sample	Control	Seaweed Inclusion Levels		
		1%	2.5%	5%
Pork oyster	45.00	44.00	42.50	40.00
Pork back fat	20.00	20.00	20.00	20.00
Water	20.00	20.00	20.00	20.00
Rusk	12.50	12.50	12.50	12.50
Seasoning	2.50	2.50	2.50	2.50
Seaweed	0.00	1.00	2.50	5.00

**Table 2:** Proximate composition, salt, WHC, and cook loss of control sausage and sausages containing 1%, 2.5%, and 5% brown and red seaweed species.

Treatment		Protein	Moisture	Fat	Ash	Salt	WHC	Cook Loss
Control		14.09 ± 0.47 ^a^	54.46 ± 0.89 ^abc^	20.02 ± 0.58 ^a^	2.23 ± 0.04 ^a^	1.47 ± 0.10 ^a^	63.73 ± 1.62 ^ab^	29.45 ± 0.79 ^abc^
SS	1%	13.14 ± 0.29 ^a^	55.71 ± 0.75 ^c^	19.65 ± 0.19 ^a^	2.40 ± 0.07 ^a^	1.61 ± 0.03 ^a^	69.25 ± 1.96 ^bdef^	30.27 ± 1.72 ^abc^
	2.5%	13.72 ± 0.51 ^a^	55.63 ± 0.22 ^bc^	19.14 ± 0.53 ^a^	2.85 ± 0.05 ^bc^	1.77 ± 0.16 ^abc^	70.96 ± 3.48 ^def^	28.57 ± 0.80 ^abc^
	5%	14.31 ± 1.21 ^a^	54.02 ± 1.65 ^abc^	18.79 ± 0.33 ^a^	3.39 ± 0.23 ^de^	2.10 ± 0.01 ^bcd^	74.95 ± 1.36 ^f^	25.05 ± 2.37 ^a^
IW	1%	13.61 ± 0.17 ^a^	55.03 ± 0.41 ^abc^	19.55 ± 0.29 ^a^	2.50 ± 0.02 ^ab^	1.61 ± 0.05 ^a^	67.47 ± 1.50 ^bde^	31.98 ± 2.01 ^bc^
	2.5%	13.48 ± 0.36 ^a^	54.32 ± 0.71 ^abc^	19.29 ± 0.42 ^a^	3.02 ± 0.10 ^cd^	1.86 ± 0.13 ^abcd^	71.18 ± 0.48 ^def^	30.52 ± 5.48 ^abc^
	5%	13.84 ± 0.63 ^a^	54.89 ± 1.00 ^abc^	18.99 ± 0.42 ^a^	3.66 ± 0.02 ^e^	2.26 ± 0.19 ^d^	73.20 ± 2.58 ^ef^	29.97 ± 2.49 ^abc^
PP	1%	13.38 ± 0.13 ^a^	53.82 ± 1.34 ^abc^	19.69 ± 0.70 ^a^	2.48 ± 0.04 ^ab^	1.60 ± 0.29 ^a^	55.05 ± 1.92 ^c^	33.23 ± 0.45 ^c^
	2.5%	14.38 ± 0.62 ^a^	53.52 ± 0.64 ^abc^	19.25 ± 0.20 ^a^	2.90 ± 0.06 ^bc^	1.80 ± 0.19 ^abc^	60.66 ± 0.46 ^ac^	31.04 ± 0.72 ^abc^
	5%	14.51 ± 1.01 ^a^	53.13 ± 0.42 ^ab^	18.79 ± 0.22 ^a^	3.67 ± 0.44 ^e^	2.21 ± 0.10 ^cd^	64.98 ± 2.43 ^ab^	27.38 ± 0.69 ^abc^
Nori	1%	14.04 ± 0.34 ^a^	53.43 ± 0.29 ^abc^	19.88 ± 0.20 ^a^	2.38 ± 0.04 ^a^	1.47 ± 0.08 ^a^	66.14 ± 2.66 ^abd^	28.29 ± 2.25 ^abc^
	2.5%	14.43 ± 0.36 ^a^	52.81 ± 0.74 ^a^	19.45 ± 0.69 ^a^	2.54 ± 0.13 ^ab^	1.62 ± 0.19 ^a^	69.30 ± 1.04 ^bdef^	26.14 ± 0.73 ^ab^
	5%	15.60 ± 0.88 ^a^	53.09 ± 0.86 ^ab^	18.90 ± 0.12 ^a^	3.17 ± 0.10 ^cd^	1.73 ± 0.17 ^ab^	73.36 ± 2.06 ^ef^	25.76 ± 1.74 ^ab^

^abcdef^ % Mean values (±standard deviation) in the same column bearing different superscripts are significantly different (*p* < 0.05).

**Table 3:** CIE L* a* b* values of control sausage and sausages containing 1%, 2.5%, and 5% brown and red seaweed species.

Treatment		L*	a*	b*
Control		70.45 ± 0.25 ^a^	6.25 ± 0.16 ^a^	13.87 ± 0.29 ^a^
SS	1%	66.54 ± 3.33 ^abc^	4.29 ± 1.08 ^bc^	13.35 ± 0.53 ^ab^
	2.5%	64.90 ± 1.96 ^abcd^	2.97 ± 0.15 ^cde^	13.16 ± 0.30 ^ab^
	5%	61.73 ± 3.39 ^bcde^	1.42 ± 1.05 ^e^	12.77 ± 1.12 ^abc^
IW	1%	66.15 ± 0.90 ^abc^	2.05 ± 0.96 ^de^	12.39 ± 0.71 ^abc^
	2.5%	59.71 ± 2.22 ^de^	−0.65 ± 0.26 ^f^	10.90 ± 0.71 ^bcd^
	5%	53.29 ± 2.44 ^fg^	−2.01 ± 0.20 ^f^	9.55 ± 0.93 ^de^
PP	1%	67.78 ± 2.64 ^ab^	3.94 ± 0.58 ^bcd^	11.62 ± 0.94 ^abcd^
	2.5%	62.21 ± 0.47 ^bcd^	3.04 ± 0.46 ^cde^	9.44 ± 0.65 ^de^
	5%	59.13 ± 1.64 ^def^	2.54 ± 1.00 ^cde^	8.03 ± 1.54 ^ef^
Nori	1%	61.09 ± 1.71 ^cde^	5.29 ± 0.37 ^ab^	10.34 ± 1.53 ^cde^
	2.5%	56.05 ± 1.72 ^ef^	4.19 ± 0.59 ^bc^	8.22 ± 0.63 ^ef^
	5%	48.96 ± 2.02 ^g^	2.70 ± 0.33 ^cde^	6.19 ± 0.70 ^f^

^abcdef^ % Mean values (±standard deviation) in the same column bearing different superscripts are significantly different (*p* < 0.05).

**Table 4:** TPA of control sausage and sausages containing 1%, 2.5%, and 5% brown and red seaweed species.

Treatment		Hardness (N)	Adhesiveness (N)	Springiness (mm)	Cohesiveness	Chewiness (N*mm)
Control		42.81 ± 3.47 ^ab^	−0.022 ± 0.02 ^a^	0.862 ± 0.02 ^abc^	0.664 ± 0.04 ^abc^	24.41 ± 2.05 ^abc^
SS	1%	35.67 ± 1.83 ^ab^	−0.009 ± 0.01 ^a^	0.893 ± 0.03 ^ab^	0.686 ± 0.01 ^ab^	21.80 ± 1.09 ^ab^
	2.5%	45.23 ± 6.11 ^ab^	−0.012 ± 0.01 ^a^	0.903 ± 0.00 ^a^	0.669 ± 0.01 ^abc^	27.32 ± 3.94 ^bc^
	5%	46.62± 9.07 ^ab^	−0.011 ± 0.00 ^a^	0.843 ± 0.04 ^abcd^	0.704 ± 0.02 ^a^	27.76 ± 6.34 ^bc^
IW	1%	41.49 ± 1.62 ^ab^	−0.009 ± 0.00 ^a^	0.891 ± 0.02 ^ab^	0.671 ± 0.01 ^abc^	24.89 ± 1.26 ^abc^
	2.5%	44.95 ± 3.36 ^ab^	−0.010 ± 0.00 ^a^	0.852 ± 0.01 ^abc^	0.664 ± 0.03 ^abc^	25.38 ± 3.05 ^abc^
	5%	64.13± 3.62 ^cd^	−0.021 ± 0.01 ^a^	0.813 ± 0.04 ^bcd^	0.620 ± 0.03 ^c^	32.13± 3.24 ^cd^
PP	1%	50.07 ± 6.32 ^bc^	−0.012 ± 0.01 ^a^	0.819 ± 0.03 ^abcd^	0.618 ± 0.01 ^c^	25.11 ± 2.59 ^abc^
	2.5%	41.18 ± 4.76 ^ab^	−0.022 ± 0.02 ^a^	0.783 ± 0.03 ^cd^	0.639 ± 0.03 ^bc^	20.58 ± 2.07 ^ab^
	5%	34.20 ± 2.21 ^a^	−0.035 ± 0.01 ^a^	0.759 ± 0.03 ^d^	0.650 ± 0.02 ^abc^	16.91 ± 0.78 ^a^
Nori	1%	46.20 ± 2.18 ^ab^	−0.020 ± 0.01 ^a^	0.871 ± 0.02 ^ab^	0.672 ± 0.02 ^abc^	27.10 ± 2.14 ^bc^
	2.5%	48.29 ± 5.74 ^ab^	−0.022 ± 0.01 ^a^	0.825 ± 0.05 ^abcd^	0.682 ± 0.01 ^abc^	27.20 ± 3.62 ^bc^
	5%	68.60 ± 6.61 ^d^	−0.013 ± 0.00 ^a^	0.880 ± 0.02 ^ab^	0.623 ± 0.01 ^bc^	37.42 ± 2.82 ^d^

^abcd^ % Mean values (±standard deviation) in the same column bearing different superscripts are significantly different (*p* < 0.05).

**Table 5:** Sensory (hedonic) scores of control sausage and sausages containing 1%, 2.5%, and 5% brown and red seaweed species.

Treatment		Appearance	Aroma	Texture	Flavour	Overall Acceptability
Control		6.78 ± 1.76 ^ab^	6.87 ± 1.46 ^ab^	5.72 ± 2.07 ^ab^	6.70 ± 1.69 ^ab^	6.78 ± 1.76 ^ab^
SS	1%	7.18 ± 1.20 ^a^	6.89 ± 1.48 ^ab^	6.53 ± 1.46 ^a^	7.39 ± 1.59 ^a^	7.27 ± 1.45 ^a^
	2.5%	6.63 ± 1.02 ^ab^	5.23 ± 2.03 ^bcd^	5.71 ± 1.51 ^ab^	5.04 ± 2.09 ^bcd^	5.23 ± 1.71 ^bcde^
	5%	5.78 ± 2.03 ^abc^	4.12 ± 2.41 ^d^	5.07 ± 2.01 ^ab^	3.14 ± 2.69 ^d^	3.81 ± 2.50 ^e^
IW	1%	6.37 ± 1.13 ^ab^	6.82 ± 1.22 ^ab^	5.76 ± 1.96 ^ab^	6.68 ± 1.28 ^ab^	6.26 ± 1.30 ^abc^
	2.5%	5.24 ± 1.87 ^bc^	5.38 ± 1.85 ^bcd^	5.65 ± 1.96 ^ab^	5.32 ± 1.84 ^abc^	5.55 ± 1.63 ^abcde^
	5%	3.19 ± 1.99 ^d^	4.42 ± 1.97 ^cd^	4.37 ± 1.58 ^b^	4.38 ± 2.41 ^cd^	4.12 ± 2.08 ^de^
PP	1%	6.96 ± 1.65 ^ab^	7.23 ± 1.16 ^a^	5.84 ± 1.98 ^ab^	5.83 ± 2.08 ^abc^	5.91 ± 1.77 ^abcd^
	2.5%	6.03 ± 1.82 ^abc^	6.05 ± 1.67 ^abc^	5.29 ± 2.20 ^ab^	4.34 ± 0.61 ^cd^	4.78 ± 2.79 ^cde^
	5%	5.44 ± 1.37 ^abc^	4.79 ± 2.25 ^cd^	5.01 ± 2.05 ^ab^	3.23 ± 2.28 ^d^	4.17 ± 2.14 ^de^
Nori	1%	7.01 ± 1.36 ^a^	6.87 ± 1.40 ^ab^	5.71 ± 1.63 ^ab^	7.08 ± 1.57 ^ab^	6.98 ± 1.60 ^ab^
	2.5%	6.23 ± 2.00 ^abc^	6.87 ± 1.23 ^ab^	5.35 ± 2.25 ^ab^	6.82 ± 1.63 ^ab^	6.23 ± 1.71 ^abc^
	5%	4.60 ± 2.22 ^cd^	6.02 ± 1.17 ^abc^	5.44 ± 1.73 ^ab^	6.58 ± 1.99 ^ab^	6.18 ± 1.84 ^abc^

^abcde^ Mean values (±standard deviation) in the same column bearing different superscripts are significantly different (*p* < 0.05).

## Data Availability

The data presented in this study are available in article.
